# Reward boosts cognitive control during working memory maintenance

**DOI:** 10.1038/s41598-025-09949-1

**Published:** 2025-08-11

**Authors:** Béla Weiss, Annamária Manga, Ádám Nárai, Adél Bihari, Judit Zsuga, Zoltán Vidnyánszky

**Affiliations:** 1 Brain Imaging Centre, HUN-REN Research Centre for Natural Sciences, Budapest, Hungary; 2 Department of Cognitive Science, Faculty of Natural Sciences, Budapest University of Technology and Economics, Budapest, Hungary; 3 Department of Psychiatry, Faculty of Medicine, University of Debrecen, Debrecen, Hungary

**Keywords:** Working memory, Pupil dilation, Reward, Cognitive control, Reward, Cognitive control, Human behaviour, Working memory

## Abstract

Working memory (WM) involves short-term maintenance and manipulation of goal-relevant information, with cognitive control playing a crucial role in these processes due to WM’s limited capacity. Pupillometry studies show distinct pupillary changes for WM stages, reflecting cognitive effort and load. Motivational incentives enhance WM performance by potentially improving encoding, maintenance, or retrieval, though the specific components influenced by reward remain unclear. This study specifically tested whether reward modulates cognitive control processes during WM maintenance using pupillometry. Participants performed a delayed-estimation orientation WM task with reward cues indicating reward levels at the beginning of trials. The results revealed that motivational incentives significantly improved WM performance and increased pupillary dilation during maintenance. These findings provide evidence for the modulation of WM maintenance by reward through enhanced top-down cognitive control processes.

## Introduction

Working memory (WM) is a fundamental cognitive construct coined to describe the ability to encode, maintain, manipulate and retrieve goal-relevant information. Individual differences in WM performance seem to account for differences in higher cognitive processes such as learning, reading comprehension, fluid reasoning and scholastic aptitude^1^. This shared variance may stem from one common factor, e.g. cognitive control, as the faculty to select and activate goal-relevant information in light of uncertainty and distractions is an important common factor underscoring WM and higher-level cognitive performance^2^. The paramount significance of cognitive control seems even more straightforward if the limited capacity of WM is considered^3^.

The net of information that may be held on-line in a prioritized state seems to amount to four separate items^4^. This well-defined capacity limit mandates that goal relevant, rather than distracting information is attended, encoded, maintained and prioritized in WM during delays and task execution. This is an active effortful process that mandates the allocation of attention^5^ and is subserved by cognitive control^2^.

Prior reports have suggested that cognitive control and attentional function interact to resolve conflict in service of efficient information utilization. Attentional control enhances optimal task performance if task-relevant information is accessible and unwanted information is suppressed^6^, and by selecting the relevant subset of sensory information cognitive control may override the limited capacity of information processing^2^.

The basic function of WM is to store prior information to govern future behavior^7^. To do so, encoding takes place to create an internal representation of attended information. This representation is to be maintained for future manipulations. Subsequently, task-relevant content is prioritized and maintained content is transformed to optimize impeding behavior^8,9^. These distinct operations of (visual) WM, e.g. encoding, maintenance and prioritization also manifest in dynamic pupillary changes. Baseline pupil size captures arousal, the backdrop for encoding, and subsequent change of pupillary diameter upon stimulus presentation may signal the extent of attention allocation. Holding encoded information in WM during the maintenance phase is accompanied by pupillary dilation, the extent of which correlates with the number of items held in memory. Prioritization is accompanied by further pupillary dilation^10^.

Previous work has suggested that motivational incentives could increase WM capacity per se, by enhanced encoding of all items^11,12^. Others suggested that improved temporal maintenance by reduced drift and decay of memories^13^ or better recovery of items due to increased accuracy of decision processes^14^ could be involved. Nevertheless, involvement of attentional control in WM must also be addressed in the context of strategic prioritization of items. It was shown that by allocating differential rewards for correct responses, differential distribution of value may be deduced with a primacy–recency asymmetry. This could indicate that limited capacity storage and processing may be flexibly alternated with respect to allocated value^6^.

Albeit, the effect of reward on WM has been established previously, the precise mechanism regarding which component of WM is influenced by reward is yet to be elucidated. A recent study, using verbal complex span task followed by delayed recall test assessed whether manipulation of cognitive load or applying monetary incentives influences WM maintenance. The authors revealed improved results on delayed recall yet attributed this to combined change in encoding and maintenance, and set forward the need to further elucidate the nature of this modulation^15^.

Additional to serving as an index for cognitive effort and cognitive load^16^ emerging evidence points to pupil size being also responsive to affective influences such as motivation and reward^17^. Thus, by being at the interface of motivation and cognition, pupillometry seems suitable to assess the dynamic interactions between these domains, detection of the pupillary response may help to pinpoint and tease apart the components of WM that are influenced by reward.

Here, using pupillometry, we investigated the modulation of cognitive control during WM maintenance by reward application. Using a modified, incentivized version of a delayed-estimation task, participants were asked to memorize the color and orientation of bars and adjust the orientation of probes to match that of the target bar. Reward collection commenced from the half of the experiment, with symbolic reward cues indicating the reward level of trials (low or high reward). Participants’ WM performance was characterized by the absolute recall error, parallel to the estimation of baseline-corrected pupillary area during WM maintenance. In addition, the behavior of participants was assessed also by measuring their reaction time. We contrasted the behavioral and pupil size measures between trials with and without reward, and also assessed the effects of reward level. Our main hypothesis was that reward level should affect all three analyzed measures, higher reward should be associated with better WM performance, i.e. lower absolute recall error, larger pupil size and longer reaction time compared to lower reward. Moreover, we also tested the potential modulation of reward effects over time to assess whether the time spent on task and fatigue due to sustained cognitive activity affects the investigated processes. Namely, the time on task, tiredness and lower level of alertness may also influence behavior and associated measures, including the pupil diameter. Prior studies have depicted an inverse relationship between cognitive fatigue and accuracy indicated by a rise in errors as fatigue accumulates, and pupil size has been shown to decrease with time on task, an effect that could be compensated by applying high reward^1,18–27^. Accordingly, our hypothesis was if time-on-task and cognitive-fatigue effects arise, drop in behavioral performance and decrease of pupil size should occur with time. However, if longer adaptation occurs, it can compensate these effects and perhaps even enhance the participants’ performance over time.

## Methods

### Participants

Forty-four right-handed young adults were recruited to participate in the experiment, with normal or corrected-to-normal vision (including color-vision) and no reported history of neurological or psychiatric disorders. Six participants were excluded from statistical analyses based on eye-tracking data quality criteria. Thus, we report the data of 38 subjects with age between 20 and 24 years (mean age 21.29 years, standard deviation SD = 1.25 years; 17 female). The sample size was chosen based on highly relevant recent studies^5,17,28,29^. The study was approved by the National Institute of Pharmacy and Nutrition (file number: OGYÉI/70184/2017), and the experiment was conducted in accordance with relevant guidelines and regulations at the Brain Imaging Centre, HUN-REN Research Centre for Natural Sciences in Budapest, Hungary. Each subject provided written informed consent and received a monetary reimbursement (3€/hour).

### Experimental task

**Fig. 1:**
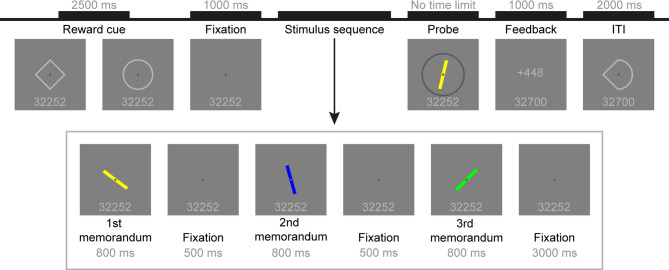
Schematic illustration of one experimental trial. After presentation of the reward cue stimulus (open diamond or circle), participants had to memorize three colored bars shown sequentially, and adjust the probe bar’s orientation using the keyboard to replicate the orientation of the target bar with the corresponding color. After the feedback, a precue stimulus was shown during the inter-trial interval (ITI).

Participants performed a modified, incentivized version of the delayed-estimation task^30^ in which during the course of a trial, participants were instructed to memorize three consecutively presented bars with different colors and orientations, and adjust the orientation of the probe bar to the memorized target bar with the matching color. The time spent on adjusting the probe bar was not limited and it is considered to be the participants’ reaction time. Participants completed 12 runs of the task, with 36 trials per run. At the beginning of each trial, a reward cue indicated whether small or large reward could be obtained in the trial. The detailed description of the task is published in a recent paper^31^. Here, we implemented this experimental paradigm with some amendments (Fig. 1). The reward level of the trial (low or high) was indicated by symbolic reward cues (empty circle or diamond, in light grey color (RGB: 180,180,180), at the center of the screen, 2° × 2° of visual angle, 0.15° line width), with counterbalanced cue-reward level mapping across the participants. The major modification is that reward collection was introduced after the completion of the sixth run. In order to preserve the trial structure, reward cues were presented in the first six runs as well, however, they were meaningless to the participants. The obtained reward (in the units of scores) in a trial was calculated from the reciprocal of the error on the current trial, multiplied by a small constant (c = 5) in low-reward, and by a large constant (c = 500) in high-reward trials. The collected score in the current trial was presented as a feedback at the end of each trial at the center of the screen, over a fixation disc, with the cumulative reward score presented under the fixation disc, both in light grey color (RGB: 180,180,180). In the first six runs, a zero under and over the fixation disc was presented as a feedback. After the feedback, a precue preceding the next reward cue was shown during the inter-trial interval (ITI). The precue was a composite of the diamond and circle, matched to the visual properties of the reward cues. The timing of visual stimuli remained, except the duration of reward cues (2500 ms) and the ITI (2000 ms). Participants completed 18 practice trials before the first run, and 12 practice trials before the seventh run to establish the meaning of the newly introduced reward cues.

Participants’ working-memory performance was characterized by the absolute recall error, the absolute circular difference between the angle of target bars and the angle that was set by subjects for the probe bars. To test whether the participants’ affective reactions (AR) to the cues representing low and high reward differ, subjects were asked to fill in a self-reported affective reaction test^32^ at the end of the experiment.

### Pupil data collection and processing

Pupil area of the right eye was recorded during the 12 experimental runs using an infrared eye-tracking camera (Eyelink 1000 Plus Tower Mount, SR Research Ltd., Mississauga, ON, Canada) in arbitrary units, at a sampling rate of 1000 Hz. Participants’ head was supported on the tower mount’s chin and forehead rest, at a viewing distance of 54 cm from the display (24” Fujitsu LED monitor, 60 Hz refresh rate). Participants were instructed to fixate on a fixation disc at the center of the screen during the runs. The recording started at the beginning of each run with a standard nine-point calibration procedure, and ended at the end of the run. Participants did not receive any specific instruction on blinking. Preprocessing of pupil area data was performed at run level. Blinks (zero values in the data) and saccades (as detected by the default algorithm and settings of the eye-tracking software) with 100 ms windows before and after the eye closures and movements were flagged. Flagged data points were replaced with linearly interpolated values, smoothed with low-pass filtering (second-order Butterworth filter with 10 Hz cut-off frequency, using the filtfilt() MATLAB function) and then standardized using the median and interquartile range statistics. In the next step, pupil area data was segmented into trials. Trials with interpolated data points exceeding 50% of the trial were excluded from the analysis. Since the window available for responding was not limited resulting in varying probe period lengths, we omitted these response periods from estimating the percentage of artefactual sample points. To be considered for statistical evaluation, experimental runs had to contain at least five remaining trials per reward condition. Participants with less than 10 remaining runs (*N* = 6) were excluded from statistical analysis. Data were visually inspected at this point to confirm that artefacts had been properly handled. In order to investigate the effects of reward on pupil size during WM maintenance, we statistically evaluated the last 1.5 s of the delay period (the 3s-long time interval between the offset of the last target stimulus bar and the onset of the probe bar). The first 1.5 s of the delay period was not considered due to transients occurring after the offset of the last target stimuli. Variation of pupil size during WM maintenance was considered as a phasic activity starting from the onset of the delay period, similarly to task-evoked pupillary responses (TEPRs) during which the pupil size changes relative to tonic baseline levels due to fluctuations in cognitive processing load^1^. In order to control for potential confounding effects originating from variation of baseline pupil area across the experimental runs and trials, we applied subtractive baseline correction. We defined the baseline as the mean of the last 200 ms before the onset of the delay period and corrected each trial by the trial’s own baseline value. Baseline-corrected pupil area was averaged within the selected time interval at trial level and then averaged across experimental trials and runs. Pupil data was processed using custom-written scripts in MATLAB R2013b (MathWorks, Natwick, MA).

### Statistical analyses

Participants’ affective reactions to low- and high-reward cue stimuli were compared by a paired samples t-test, while the effects of binning the experimental runs and the type of reward cues (low and high reward) on WM performance, reaction time and TEPR-like baseline corrected pupil area were tested by two-way repeated measures ANOVA tests. Assumptions of ANOVA tests were evaluated by also considering the Greenhouse-Geisser corrected P values, and multiple comparisons correction was applied to post-hoc test results using the Benjamini and Hochberg approach for controlling the false discovery rate (FDR^33^). Hypothesis testing was carried out without and with outlier correction. Outlier values were detected using the interquartile rule. Lower threshold was defined as Q_1_-1.5×IQR while the upper threshold was Q_3_ + 1.5×IQR, where Q_1_ and Q_3_ were the first and third quartiles, respectively, and the interquartile range was IQR = Q_3_-Q_1_. Data points below the lower and above the upper limits were considered outliers. Participants were marked for exclusion if outlier value was found in any combination of bins and reward cues. Although the same effects were found for both hypothesis test approaches, here, we provide the results obtained after outlier elimination. Statistical analyses were performed using the pingouin (version 0.5.3) Python package^34^.

We also assessed the relationship between WM performance and pupil size as well as between reaction time and pupil size by correlation analyses. More specifically, we tested the relationship between behavioral measures and pupil area in the last 1.5s of the delay period by using normalized measure differences ((H-L)/(abs(H) + abs(L))), where H and L indicate individual averages across trials with high- and low-reward cue stimuli, respectively. Besides running the analysis for the second half of the experiment when reward was applied (bin REW), for validation purposes, the correlations were tested also for the first half of the experiment when participants were not aware of the meaning of reward cues (bin NREW). Spearman correlations were calculated without and with outlier elimination and correction for multiple correlations (REW and NREW). Skipped Spearman correlations and their 97.5% confidence intervals (CI) were calculated after elimination of bivariate outliers and using 1000 bootstrap iterations as implemented in the Robust Correlation Toolbox^35^.

## Results

### Behavior

Testing the effect of reward level on self-reported affective reactions revealed significantly (t(37) = 7.79, P = 2.6 × 10^− 9^, Cohen’s d’ = 1.57) higher ratings for high-reward (mean AR = 7.78, SD = 1.65) compared to low-reward cue stimuli (mean AR = 5.40, SD = 1.37), suggesting that participants understood the meaning of reward stimuli and exhibited stronger reactions to high- compared to low-reward cues in agreement with previous findings^31^.

**Fig. 2:**
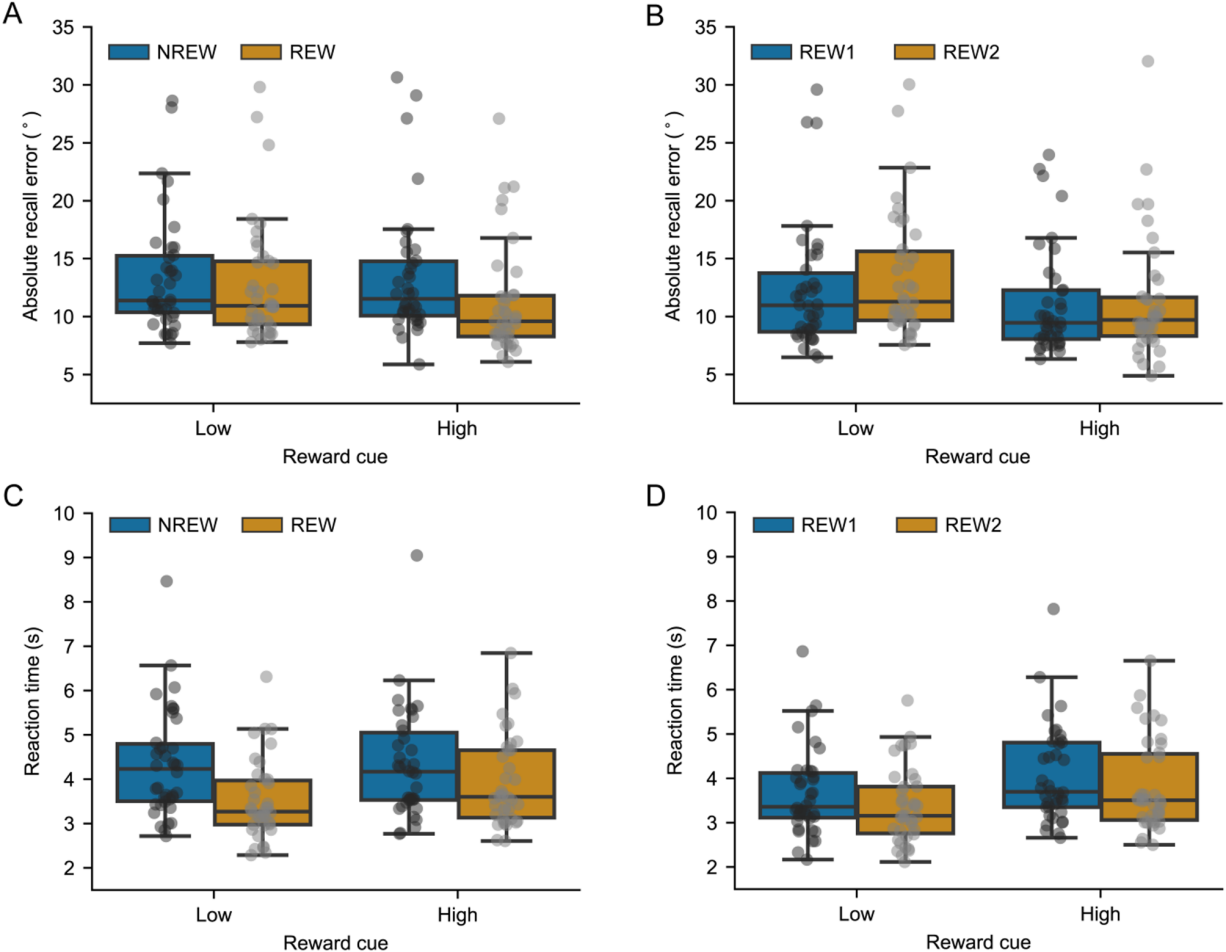
Absolute recall error and reaction time. Recall error and reaction time are provided for the first (NREW) and second (REW) halves of the experiment, indicating experimental runs without and with reward application, respectively (A and C), as well as for the first (REW1) and second (REW2) halves of experimental runs in which reward was applied (B and D). Whiskers indicate minimum and maximum values that are still within the acceptable range determined by the outlier detection procedure. Grey dots represent individual averages. Dots above whiskers indicate outlier values detected separately for each combination of bins and reward cues.

The working-memory performance, reaction time and pupil size in the delay period were evaluated by contrasting the first (without reward; NREW) and second (with reward; REW) halves of the experiment as well as by comparing the first (REW1) and second (REW2) halves of runs in which reward was applied. When experimental runs were binned according to reward application (NREW: first half of the experiment, the first six runs without reward; REW: second half of the experiment, the last six runs with reward; Fig. 2A), significant main effects of reward application (F(1,32) = 17.39, *P* = 2 × 10^− 4^, η^2^ = 0.057, number of outlier participants NO = 5) and reward level (F(1,32) = 12.39, *P* = 0.001, η^2^ = 0.029) were found for the absolute recall error WM performance metric, indicating larger error for the experimental runs without reward application and smaller error for trials with high- compared to those with low-reward cue stimuli, respectively. Furthermore, a significant interaction between the two independent variables was also obtained (F(1, 32) = 6.13, *P* = 0.02, η^2^ = 0.016). This interaction is likely to occur due to the study design as the meaning of reward cue stimuli was revealed to participants only after the last run of the NREW bin, at the mid of the experiment. Accordingly, the distinction of responses to high- and low-reward cues may be considered meaningless in the first half of the experiment (NREW). To test this assumption, post hoc analyses were carried out. Post hoc tests revealed significantly smaller recall error for trials with high- compared to low-reward cue stimuli in the second half of the experiment when reward was applied (REW: t(32)=-3.36, P_FDR_=0.004, η^2^ = 0.088), while no such a difference was found for the first half of the experiment when participants were not aware of the meaning of the reward cue symbols (NREW: t(32)=-0.97, P_FDR_=0.34, η^2^ = 0.002). Moreover, significantly better working-memory performance, i.e., smaller recall error was found for the trials with high-reward cues when the meaning of reward cues was known to the participants compared to the first half of the experiment (t(32) = 5.57, P_FDR_=10^− 5^, η^2^ = 0.13). No such an effect was found for the trials with low-reward cue stimuli (t(32) = 1.32, P_FDR_=0.20, η^2^ = 0.013).

To investigate how the effect of reward might change through time, the second half of the experimental runs (REW in previous analysis) was split into two bins (Fig. 2B), both consisting of three runs (REW1: the first three experimental runs with reward, the first half of REW; REW2: the second half of REW, the last three runs of the experiment). In this case, only a significant main effect of reward level on absolute recall error was found (F(1,31) = 13.07, *P* = 0.001, η^2^ = 0.092, NO = 6). The effect of binning (REW1 vs. REW2: F(1, 31) = 2.98, *P* = 0.094, η^2^ = 0.010) and the interaction between binning and reward level (F(1,31) = 3.83, *P* = 0.06, η^2^ = 0.011) were not significant. As the P value of interaction was close to the significance level, post hoc testing was also carried out. Post hoc testing revealed significantly smaller recall error in the high- compared to low-reward condition in both first (REW1: t(31)=-2.13, P_FDR_=0.041, η^2^ = 0.045) and second halves (REW: t(31)=-3.83, P_FDR_=0.001, η^2^ = 0.141) of trials with reward. In the low-reward condition, the performance was significantly better in the first (REW1) compared to the second half (REW2) of runs with reward (t(31)=-2.39, P_FDR_=0.046, η^2^ = 0.035), while no such a difference was found for trials with high reward (t(31) = 0.10, P_FDR_=0.92, η^2^ = 8 × 10^− 5^).

For the reaction time (Fig. 2C), significant main effect of reward application (F(1,36) = 25.37, *P* = 10^− 5^, η^2^ = 0.078, NO = 1) and reward-level cue stimulus (F(1,36) = 24.98, *P* = 2 × 10^− 5^, η^2^ = 0.012) was found along with significant interaction between the two independent variables (F(1,36) = 31.81, *P* < 10^− 5^, η^2^ = 0.015). Post hoc testing revealed significantly longer reaction time for trials with high- compared to low-reward cue stimuli (t(36) = 5.75, *P*_*FDR*_ < 10^− 5^, η^2^ = 0.059) in the second half of the experiment when reward was applied, and no such a difference was found for the first half of the experimental trials without reward application (NREW: t(36)=-0.73, P_FDR_=0.47, η^2^ = 1.5 × 10^− 4^). Furthermore, the reaction time was significantly longer in the first compared to the second half of the experiment for both high (t(36) = 2.70, P_FDR_=0.01, η^2^ = 0.025) and low-reward (t(36) = 6.63, P_FDR_<10^− 5^, η^2^ = 0.158) cue stimuli. When considering the binning of trials with reward only (second half of the experiment, bins REW1 and REW2, Fig. 2D), significant main effects of binning (F(1,35) = 14.39, *P* = 5.6 × 10^− 4^, η^2^ = 0.016, NO = 2) and reward-cue stimulus (F(1,35) = 33.32, *P* < 10^− 5^, η^2^ = 0.058) was found without interaction between the two variables (F(1,35) = 0.76, *P* = 0.39, η^2^ = 5.3 × 10^− 4^). The reaction time was longer for high compared to low reward in both first (REW1: t(35) = 4.62, P_FDR_=5 × 10^− 5^, η^2^ = 0.052) and second (REW2: t(35) = 5.13, P_FDR_=2 × 10^− 5^, η^2^ = 0.064) halves of trials with reward. The participants reacted faster in the second (REW2) compared to the first (REW1) half of trials with reward considering both high- (t(35) = 2.40, P_FDR_=0.022, η^2^ = 0.009) and low-reward (t(35) = 3.53, P_FDR_=0.002, η^2^ = 0.027) trials.

### Pupil size

**Fig. 3:**
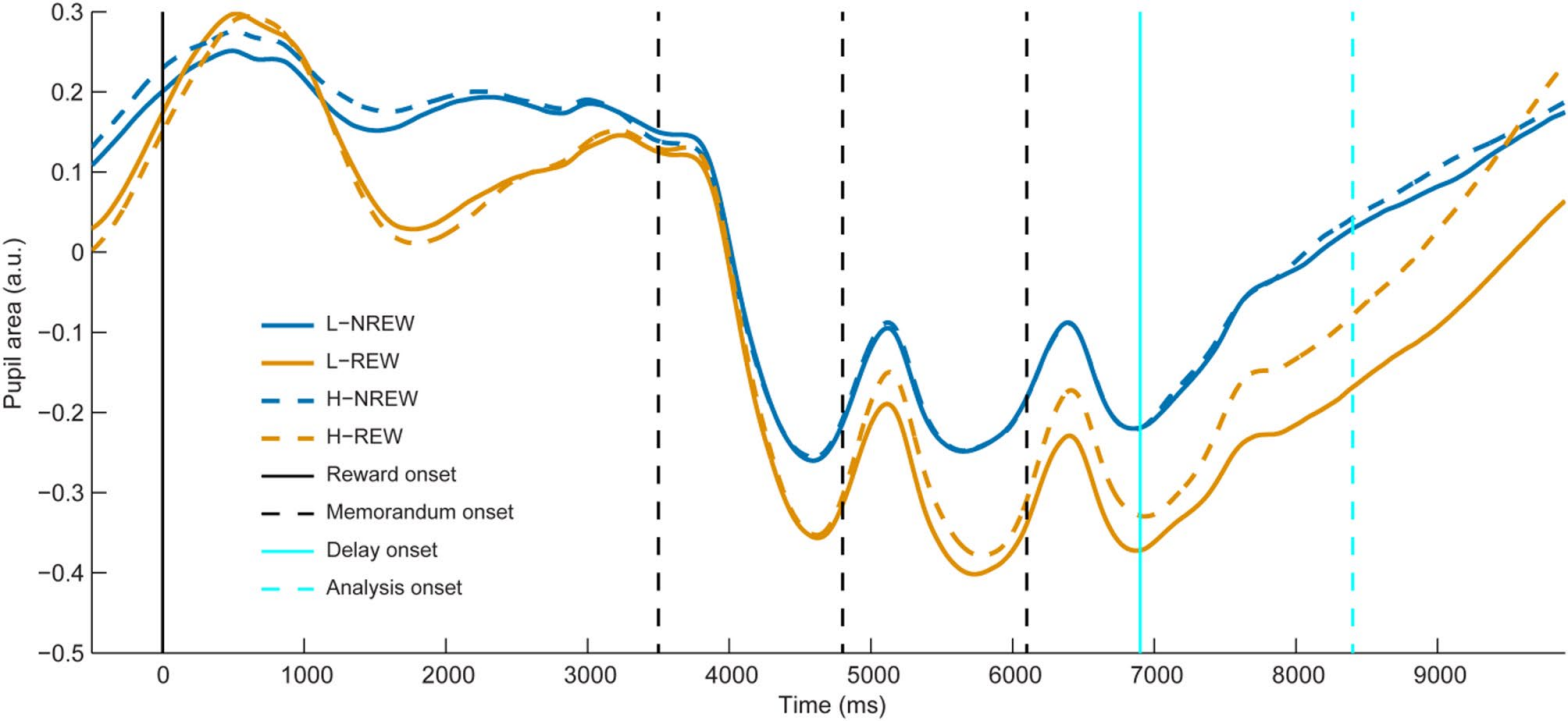
Grand average standardized pupil area without baseline correction. Pupil area is provided separately for low (L) and high (H) reward cue stimuli from 500 ms before the onset of reward cues (solid vertical black line) till the end of the delay period. The experimental runs were binned, the first half of runs, the runs without reward application are denoted by NREW, while the second half of runs, the runs with reward are indicated by REW. The dashed vertical black lines indicate the onset of memorandum stimuli, while the solid vertical cyan line denotes the onset of the delay interval. The dashed vertical cyan line marks the mid of the delay period and the beginning of the time window used for statistical analyses. Statistical analyses were performed on pupil data averaged in the last 1.5 s of the delay period, in the time window between the dashed cyan vertical line and the end of the delay interval.

**Fig. 4:**
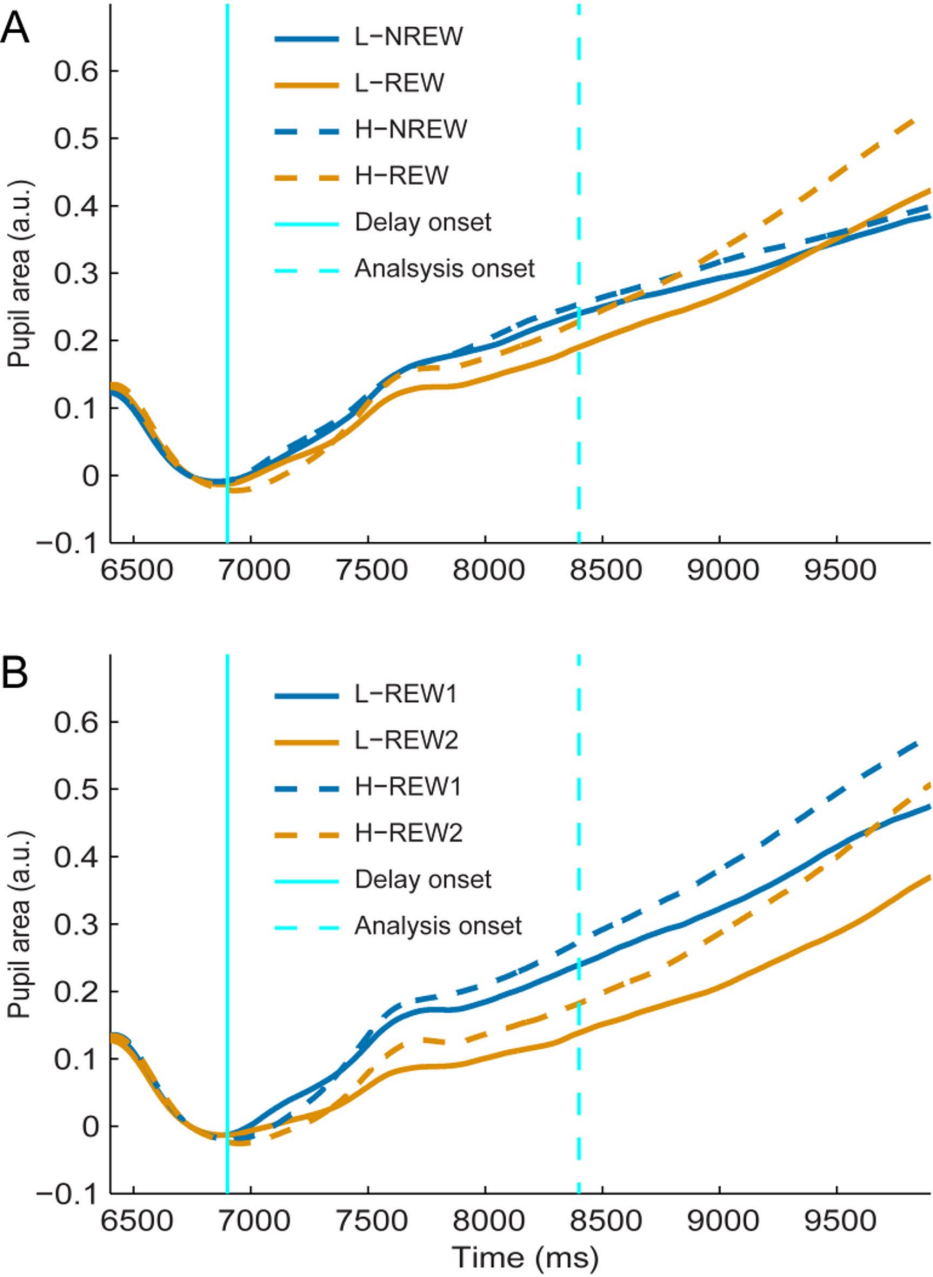
Grand average standardized and baseline corrected pupil area during the delay period. Pupil area is provided separately for low (L) and high (H) reward cue stimuli from 500 ms before the onset of the delay period (solid vertical cyan line) till its end. The experimental runs were binned in two ways. In the upper panel (A), the first half of the experimental runs, the runs without reward application are denoted by NREW, while the second half of runs, the runs with reward are indicated by REW. In the lower panel (B), only the runs with reward were considered and split into two bins each containing three runs (REW1: runs 7–9, the first three runs with reward; REW2: runs 10–12, the last three experimental runs). The dashed vertical cyan line indicates the mid of the delay period and the beginning of the time window used for statistical analyses. Statistical analyses were performed on pupil data averaged in the last 1.5 s of the delay period, in the time window between the dashed cyan vertical line and the end of the delay interval.

Visual inspection of pupil size without baseline correction indicated a sustained shift between the two halves of the experiment already before the onset of the delay period (Fig. 3). To control for this variation of pupil area and focus the statistical analyses on investigating the effects of reward on pupil dilation in the delay period, pupil area was baseline corrected (Fig. 4).

**Fig. 5:**
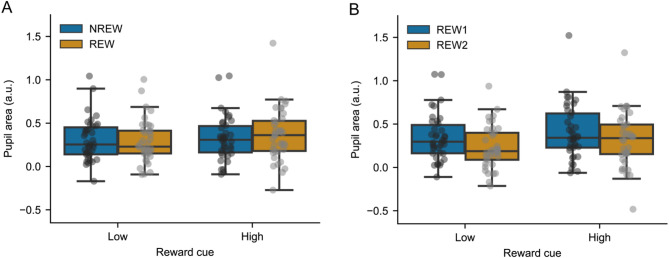
Standardized and baseline-corrected pupil area in the last 1.5 s of the delay period. Pupil area is provided for the first (NREW) and second (REW) halves of the experiment, indicating experimental runs without and with reward application, respectively (A), as well as for the first (REW1) and second (REW2) halves of experimental runs in which reward was used (B). Whiskers indicate minimum and maximum values that are still within the acceptable range determined by the outlier detection procedure. Grey dots represent individual averages. Dots above and below whiskers indicate outlier values detected separately for each combination of bins and reward cues.

When experimental runs were binned according to the application of reward (NREW and REW; Figs. 4A and 5A), a significant main effect on baseline-corrected pupil area in the delay period was found for reward level (low vs. high reward cue: F(1, 34) = 11.42, *P* = 0.002, η^2^ = 0.011, NO = 3), but not the application of reward (NREW vs. REW: F(1, 34) = 0.18, *P* = 0.67, η^2^ = 6.5 × 10^− 4^). The main effects were qualified by the significant interaction (F(1, 34) = 4.31, *P* = 0.045, η^2^ = 0.004) found between reward application and reward cue factors. Post hoc testing revealed significantly larger pupil area for trials with high- compared to those with low-reward cues when reward was applied (REW: t(34) = 3.58, P_FDR_=0.001, η^2^ = 0.025), while this difference was not significant for the first half of the experiment when participants were not aware of the meaning of the reward cues (NREW: t(34) = 0.96, P_FDR_=0.35, η^2^ = 0.002). The baseline-corrected pupil area differed between the two halves of the experiment (NREW vs. REW) neither for low-reward (t(34) = 0.77, P_FDR_=0.45, η^2^ = 0.002) nor for high-reward (t(34)=-1.14, P_FDR_=0.45, η^2^ = 0.007) cues.

Considering the experimental runs with reward only (REW1 and REW2; see above, Figs. 4B and 5B), there was no significant interaction between binning and reward level on pupil area (F(1,34) = 0.65, *P* = 0.43, η^2^ = 6.8 × 10^− 4^, NO = 3), while the main effect of binning (F(1,34) = 13.69, *P* = 7.6 × 10^− 4^, η^2^ = 0.039) and reward level (F(1,34) = 19.32, *P* = 10^− 4^, η^2^ = 0.028) was significant. The main effect of binning suggested stronger pupil dilation in REW1 compared to REW2, and this was supported by post hoc test results indicating significantly larger pupil area in REW1 compared to REW2 for both low (t(34) = 4.10, P_FDR_=4.8 × 10^− 4^, η^2^ = 0.059) and high (t(34) = 2.47, P_FDR_=0.019, η^2^ = 0.026) reward cues. The main effect of reward level indicated stronger pupil dilation for high compared to low reward that was confirmed by post hoc tests showing significantly larger pupil area for high- compared to low-reward condition during both first (REW1: t(34) = 2.69, P_FDR_=0.011, η^2^ = 0.02) and last (REW2: t(34) = 4.10, P_FDR_=4.9 × 10^− 4^, η^2^ = 0.039) three runs with reward.

Considering the relationship between the normalized absolute recall error and pupil area measures without any corrections applied, a significant Spearman correlation (r_S_=-0.38, *P* = 0.02) was obtained for trials with reward (REW), while no significant correlation (r_S_=-0.15, *P* = 0.36) was found for the first half of the experiment (NREW) when participants were not aware of the meaning of reward cue stimuli yet. The negative sign of the correlation indicates larger pupil area in the high (H) compared to low (L) reward condition in participants with lower absolute recall error in the high (H) compared to low (L) reward trials. However, none of the correlations were significant (NREW: r_S_=-0.08, *P* = 0.64, 97.5% CI=[-0.45 0.34], NO = 4; REW: r_S_=-0.31, *P* = 0.09, 97.5% CI=[-0.63 0.10], NO = 6) after eliminating the outliers and correcting for multiple correlations. Considering the relationship between the reaction time and pupil area, significant correlations were found neither without (NREW: r_S_=-0.03, *P* = 0.84; REW: r_S_=-0.02, *P* = 0.91) nor with outlier elimination (NREW: r_S_=-0.08, *P* = 0.67, NO = 3; REW: r_S_=-0.04, *P* = 0.84, NO = 3).

## Discussion

The goal of the present study was to examine the modulatory effects reward confers on visual WM performance and pupil size during WM maintenance in an incentivized delayed-estimation paradigm. WM performance and parallel change of pupillary area during WM maintenance was assessed under no reward vs. reward conditions, with further evaluations made regarding low vs. high gain states. A similar pattern of reward effects was revealed for WM performance and pupil size. Namely, increased WM performance was accompanied with stronger pupillary dilation in trials with high compared to low anticipated reward, an effect that was absent in the initial six experimental runs when reward cues were meaningless. Participants’ reaction time was longer for high compared to low reward in agreement with our previous finding^31^ indicating an increased effort to perform well on trials offering high reward. No such a difference was found between the two cues while their meaning was not known to the participants despite the shorter responses found for both reward cues when reward was applied compared to the first half of the experiment without reward application, the latter finding being in line with earlier results showing shortening of reactions by reward^36^. Moreover, investigating how the reward level effects might change with time spent on task revealed sustained modulation of WM performance, reaction time and pupil area by monetary incentives.

The suggested link between WM performance and pupil size was also indicated by correlation analyses exhibiting a weak relationship between normalized difference measures of absolute recall error and pupil size for trials in which reward was applied (REW). However, as the obtained significant correlation did not survive the corrections, this result should be taken into account with caution and further studies employing a larger sample size would have to be carried out to validate this tendency. Furthermore, although the other investigated behavioral measure, the reaction time was also modulated by reward application and reward level, it did not indicate any relationship with the pupil size extracted from the time interval of WM maintenance. Although the longer reaction times and higher WM performance obtained for high- compared to low-reward trials seem to be in agreement, the discrepancy in their relationship with pupil size may stem from the experimental design imposing a time limit on WM maintenance accompanied with no such time burden on providing the responses.

Characterization of WM-related processes may exploit the phenomenon that effortful attention control during WM tasks is reflected by phasic pupillary responses^37^. Pupil diameter was shown to correlate well with the amount of actively processed information during WM tasks and attentional control^38^ similar to our findings in this current study. This fluctuation of pupillary diameter was shown to be driven by the locus coeruleus-norepinephrine (LC-NE) system^39^. The LC is a neuromodulatory nucleus in the brainstem that has widespread noradrenergic projections throughout the brain, including the frontoparietal network (FPN), critical for WM and attention control^1,40^. In line with its distributed anatomical location, the LC-NE system seems to have general functions such as the regulation of overall arousal level and attentional control^41^. LC neurons are tonically activated, e.g. they have a baseline activity providing a backdrop of ongoing tonic firing rhythm with higher and lower tonic activity accounting for stress and drowsiness, respectively. Phasic firing activity, the transient increase of activity in response to salient stimuli, or top-down mechanisms, facilitate the selective processing of goal-relevant representations by biasing neural information processing via a local glutamate-noradrenaline interaction^42^. Phasic release of noradrenalin enhances the gain on neural activity, increases signal-to-noise ratio, thus depolarized neurons become more excited and inactive neurons become suppressed^43,44^ as opposed to low gain states, where excited neurons are less active, more inhibited neurons are less suppressed, so signal-to-noise ratio is lower^1^. Thus, the LC-NE system modulates several cortical networks, including the FPN, and is responsible for allocating attentional control that engages salient or goal-relevant stimuli while disengages irrelevant signals^44^. It follows that individuals who have better attentional control for maintaining goal-relevant information have higher WM capacity and are more successful in performing a task, especially under circumstances of high interference or distraction. Summarizing, the LC-NE system seems to play a critical role in attending to salient/novel environmental stimuli, especially in face of distractions.

Additional to the bottom-up influence of salience, top-down processes, e.g. the rewarding potential of an event is also important for attentional control and WM^45^. WM taps into the coordinated activation of distributed cortical and subcortical brain regions, with a dopamine-mediated fronto-striatal circuitry gating task-relevant stimuli considering available rewards and motivation. This effect was elegantly demonstrated in a previous incentivized delayed-estimation task, where participants were asked to memorize sequentially presented colored bars with different orientations, and reproduce the orientation of the cued memoranda as accurately as possible. The results indicated that monetary incentives have a global, beneficial effect on visual WM performance^31^.

The reciprocal innervation between LC and ventral tegmental area (VTA) offers plausible neurobiological bases for the interplay between dopaminergic reinforcement learning and noradrenergic attentional control^46^. Accordingly, goal-relevance is influenced by the opportunity for reward, allowing current goals to obtain priority, enhancing and suppressing relevant and irrelevant representations, respectively, translating into a winner-takes-more effect. VTA dopamine, by accounting for updating the contents of WM, gates inputs to the FPN with respect to goal-directed decision making under the premises of reward maximization^17,47^. Additionally, dorsal anterior cingulate cortex (dACC), the anatomical region near-ubiquitously associated with the neuroscience of cognitive control, is known to be reciprocally interconnected with both VTA and LC^48,49^. dACC regulates the effortful exertion of LC and VTA at cognitive and physical level^50,51^. This implies that top-down cognitive control may modulate arousal as well as intrinsic motivation emerging as function of LC and VTA respectively. Accordingly, these interactions between dACC, VTA and LC may govern optimal decision-making under the premises of competition between effortful choices with large rewards versus low effort choices with smaller rewards^52^.

Conversely, allocation of attention, is governed by motivation and the economics of limited cognitive resources, as costs and gains of efforts are weighted against each other^53^. Using pupillometry in a sustained attention task, the effect of reward on cognitive performance was assessed with the participation of healthy young adults^54^. The authors found reduced temporal bias on attentional deployment under rewarded conditions accompanied by larger pre- and poststimulus pupillary area, reflective of pro- and retroactive attentional processes. It was concluded that reward motivation by influencing preparatory and reactive attentional mechanisms improves overall attentional performance. Pupillometry studies further showed an increase of pupil diameter corresponding with reward and WM load^55^. The effect of reward on WM performance was also assessed by using the rewarded version of AX-continuous performance task supplemented with high-resolution pupillometry^17^. In this cognitive control task, ambiguous target probes must be identified by exploiting antecedent contextual cues which have to be maintained over a delay period. Reward incentives conferred a unique motivational influence indicated by transient pupillary dilation during maintenance paralleled by better performance. Conversely, performance-contingent reward prospects reflect the change in pupillary diameter and increase of pupil area was shown to be reflective of the greater cognitive effort related to voluntary task switching. Moreover, pupillometry may offer quantitative insight into the interplay between WM load and immediate reward, as phasic pupillary responses correlate with reward manipulations, e.g. phasic pupil dilation is more pronounced with increasing scalar value of reward^28^. For example, when considerable mental effort was required to perform a digit retention task both supraliminal and subliminal reward cues elicited pupillary dilation and increase of pupillary area was significantly greater when the scalar value of reward was increased (1 cent vs. 50 cents^56^). Summarizing, it seems that additional to salience, the reward context intervenes with attentional control and WM capacity as well as task engagement and motivated performance. Our results add to the current knowledge regarding the interaction between WM performance and reward embodied in pupillary dilation during WM maintenance paralleled by improved performance, by positing that high reward improves WM maintenance through activating noradrenergic LC neurons, a phenomenon indicated by pupil dilation. Albeit our findings point to the significance of motivational incentives with respect to enhanced WM performance due to modulated WM maintenance, further studies are warranted to shed light on the regulatory role of cognitive control in optimizing reward-related attentional processes.

Furthermore, the time course of pupil size without baseline correction in our study (Fig. 3) indicates different pupillary dynamics prior to the onset of reward cue stimuli between trials without (NREW) and with (REW) reward application. Based on prior work of others this phenomenon could be explained by a difference in preparatory attention control, e.g. intrinsic alertness and goal management processes^57–59^. In addition, a shift in pupil size can also be observed between trials with and without reward application following the onset of the first memorandum (between 3,500 and 4,800 ms post trial onset). This difference may reflect different cognitive processes including pupil orienting, a phenomenon indicative of encoding. This phase of pupillary response may indicate how robustly attention is utilized during stimulus encoding^10^. Nevertheless, although tempting to speculate that these processes are responsible for the observed shifts of pupil size before the onset of trials and after presentation of first memoranda, we refrain from investigating these processes as the current study was primarily planned to study pupil changes during WM maintenance in the delay period, and the block-design nature of reward application might be confounded by adaptation and fatigue mechanisms. The potential issue of baseline differences was addressed for investigating the pupil size correlates of WM maintenance in the delay period by applying baseline correction prior to statistical analysis.

Additional studies may also be needed to assess how the length of the maintenance period may affect the interaction between motivational incentivization and WM performance. Especially since, prior studies yielded contradictory results, with some studies reporting poorer performance parallel to longer maintenance periods^60,61^ while others failing to demonstrate such effect^62^.

Here, we also found that the effect of reward level on pupil size was present in both first and second halves of trials with reward, suggesting sustained modulation of pupil area by incentives. However, considering the change of pupil size over time, a significant decrease of pupil area was found in the second compared to the first half of these trials regardless of reward level. This finding seems to be in agreement with previous research indicating that pupil size decreases with time spent on task as participants get more tired, their alertness and arousal decline^1,20–27^. Moreover, in a related line of research, pupil dilations were found to be less prominent when participants were in disengaged attentional states^29^. Although previous studies also suggest decrease of WM performance with increase of cognitive fatigue and task disengagement, here, the downward shift in pupil size was paralleled by sustained WM performance. A marginally larger absolute recall error was found only by post-hoc analysis and only for low-reward trials in the second compared to first half of trials with reward. As no such difference was found for high-reward trials, one could speculate that high reward might have reduced or compensated the effects of time on task^21^ mitigating thus the detrimental effects of increasing fatigue and decreasing trial-level reward relative to cumulative reward. However, the lack of significant main and interaction effects suggests that this assumption should be treated with caution. In a former blood oxygen-level-dependent functional magnetic resonance imaging study including healthy young adults, mental fatigue was induced by a psychomotor vigilance task and the effect of monetary incentives was assessed on brain activity and task performance^63^. The authors found that providing an extra monetary reward increased the cognitive performance and the activity of brain regions that previously decreased due to fatigue. Finally, the general decrease of reaction time from trials without reward (NREW) to trials with reward (REW) as well as from the first half (REW1) to the second half (REW2) of trials with reward indicates that besides fatigue other processes such as adaptation may also play a role in shaping the observed trends of WM performance and pupil size. Nevertheless, it is still not clear how the amount of monetary reward, fatigue and adaptation may interact and affect WM performance, pupil dilation and brain activity in different tasks. Accordingly, further research should clarify whether the downward shift of pupil size by time revealed in our study is a precursor of cognitive fatigue that is not yet accompanied by WM performance decline or it is rather related to an increment in physical tiredness of eyes or some cognitive processes involved in processing of monetary reward. To address these questions, studies based on larger samples, using longer experiments and more demanding tasks may be necessary^21,22^.

In conclusion, the present study aimed to assess the potential influence top-down cognitive control has on WM maintenance by means of a pupillometry. Participants were asked to perform a delayed-estimation orientation WM task with reward cues indicating high vs. low reward levels at trial initiation. Our study showed significantly improved WM memory performance in response to motivational incentives, along with pupillary dilation during maintenance. These findings indicate that reward, by means of affording top-down cognitive control may modulate WM maintenance.

## Data Availability

Data supporting the findings of this study are available from the corresponding author on reasonable request.
